# Overweight and obese patients with nickel allergy have a worse metabolic profile compared to weight matched non-allergic individuals

**DOI:** 10.1371/journal.pone.0202683

**Published:** 2018-08-28

**Authors:** Mikiko Watanabe, Simonetta Masieri, Daniela Costantini, Rossella Tozzi, Francesca De Giorgi, Elena Gangitano, Dario Tuccinardi, Eleonora Poggiogalle, Stefania Mariani, Sabrina Basciani, Elisa Petrangeli, Lucio Gnessi, Carla Lubrano

**Affiliations:** 1 Department of Experimental Medicine, Section of Medical Pathophysiology, Food Science and Endocrinology, Sapienza University of Rome, Rome, Italy; 2 Department of Sensory Organs, Sapienza University of Rome, Rome, Italy; 3 Department of Endocrinology and Diabetes, University Campus Bio-Medico of Rome, Rome, Italy; Auburn University College of Veterinary Medicine, UNITED STATES

## Abstract

**Background:**

A lack of balance between energy intake and expenditure due to overeating or reduced physical activity does not seem to explain entirely the obesity epidemic we are facing, and further factors are therefore being evaluated. Nickel (Ni) is a ubiquitous heavy metal implied in several health conditions. Regarding this, the European Food Safety Authority has recently released an alert on the possible deleterious effects of dietary Ni on human health given the current levels of Ni dietary intake in some countries. Pre-clinical studies have also suggested its role as an endocrine disruptor and have linked its exposure to energy metabolism and glucose homeostasis dysregulation. Ni allergy is common in the general population, but preliminary data suggest it being even more widespread among overweight patients.

**Objectives:**

The aim of this study has been to evaluate the presence of Ni allergy and its association with the metabolic and endocrine profile in overweight and obese individuals.

**Methods:**

We have evaluated 1128 consecutive overweight and obese outpatients. 784 were suspected of being allergic to Ni and 666 were assessed for it. Presence of Ni allergy and correlation with body mass index (BMI), body composition, metabolic parameters and hormonal levels were evaluated.

**Results:**

We report that Ni allergy is more frequent in presence of weight excess and is associated with worse metabolic parameters and impaired Growth Hormone secretion.

**Conclusions:**

We confirm that Ni allergy is more common in obese patients, and we report for the first time its association with worse metabolic parameters and impaired function of the GH-IGF1 axis in human subjects.

## Introduction

The increasing epidemic of obesity and its comorbidities represents a substantial medical and economic burden according to the most recent WHO reports [1]. Calorie dense food and sedentary lifestyle frequently leading to positive energy balance do not seem to explain this phenomenon in its entirety. Therefore, research has focused on studying possible additional factors that could play a role in weight gain.

Ni is a highly allergenic ubiquitous heavy metal present in soil, tap water, common household products and utensils, cosmetics and foods of vegetable origin that has long been found to have an effect on several health outcomes [2, 3]. Preclinical studies have recently shown that Ni may play a role in energy metabolism and glucose homeostasis regulation [4–9]. Furthermore, it seems to exhibit an endocrine-disrupting activity, as *in vitro* studies have previously reported Ni induced impaired pituitary secretion of prolactin and Growth Hormone (GH) [10–12]. These data still need to be confirmed in a clinical setting but must reinforce the alert of the potential metabolic health concerns of Ni exposure.

The currently reported general population prevalence of Ni allergy is 8–18% in US and Europe, higher in southern countries such as Italy where it is estimated to be 16% [3, 13]. Ni allergy usually presents with cutaneous symptoms (Allergic Contact Dermatitis, ACD), but also as Systemic Ni Allergy Syndrome (SNAS), a condition initially hypothesized in the 70s by Christensen et al. who noted that a considerable number of patients sensitized to Ni presented with dermatitis in locations other than those which had been in contact with Ni-plated objects [14]. Nowadays, SNAS is considered to manifest with cutaneous (so-called Systemic Contact Dermatitis, or SCD) and extra cutaneous signs and symptoms (gastrointestinal, respiratory, neurological, etc.).

Interestingly, obese and overweight subjects frequently present with unspecific signs and symptoms that could be attributable to ACD and SNAS and, according to a recent report on a relatively small prevalently female overweight population, Ni allergy seems to be much more common in these subjects compared to the general population [15].

The 1994 EU Ni directive regulating the use of Ni in products that can come into contact with skin has reduced Ni allergy prevalence in countries that followed the directive such as Denmark and Sweden, proving that Ni allergy is largely attributable to Ni exposure. However, Southern European countries were not as abiding, and they therefore report higher prevalence data (approximately 16% compared to 10% of northern countries) with similar results being observed in North America, where Ni allergy continues to increase in younger and older men and women [3, 13, 16]. Moreover, the European Food Safety Authority (EFSA) has recently released an alert stating that individuals allergic to Ni are at increased risk of developing health conditions when exposed to current levels of Ni intake with food and water [17].

Given the alarmingly high prevalence of Ni allergy and obesity in the general population and the hints coming from previous preclinical studies suggesting a possible correlation between Ni and metabolic outcomes[4, 6, 7], our aim has been to investigate this in human subjects. We therefore assessed for symptoms and signs of ACD/SNAS all overweight and obese subjects being admitted to the High Specialization Center for the Care of Obesity, Sapienza University of Rome, from 2010 to 2016. Those with a suspect of Ni allergy were subjected to a Ni patch test, and their metabolic and hormonal status was evaluated.

We confirm that Ni allergy appears very common in obese patients, and we report for the first time that Ni allergy is associated with worse metabolic parameters and impaired function of the GH-IGF1 axis in human subjects.

## Patients and methods

### Subjects

1128 subjects accessing the High Specialization Center for the Care of Obesity, Sapienza University of Rome, from 2010 to 2016 were evaluated. All patients had medical history collected, physical exam and laboratory work performed (hematology, biochemistry, and eventual dynamic test) as part of the routine all patients accessing the Center undergo for initial evaluation. Patients presenting with at least two signs or symptoms compatible with SNAS or ACD such as gastrointestinal distress (bloating, meteorism, abdominal pain, belching, dyspepsia, diarrhea, constipation), systemic symptoms (fatigue, headache) and cutaneous signs (itching, rash, dermographia, urticaria) [18] were candidates to a Ni patch test. Exclusion criteria to undergo the diagnostic procedure were age <18 and >65, systemic treatment with corticosteroids, antihistamine or other immunosuppressive agent, pregnancy, topical treatment with corticosteroids on the test area in the past 14 days, and absence of written informed consent. Age exclusion criteria was selected in an effort to have a more homogeneous sample by excluding pediatric and older age. Those who were pregnant or refusing consent were excluded for ethical reasons. Other exclusion criteria were adopted as these conditions are known to be potentially interfering with patch test results. All procedures performed were in accordance with the ethical standards of the institutional and/or national research committee and with the 1964 Helsinki declaration and its later amendments or comparable ethical standards. The study was reviewed and approved by Sapienza University of Rome Ethics Committee.

### Stratification

**Fig 1:**
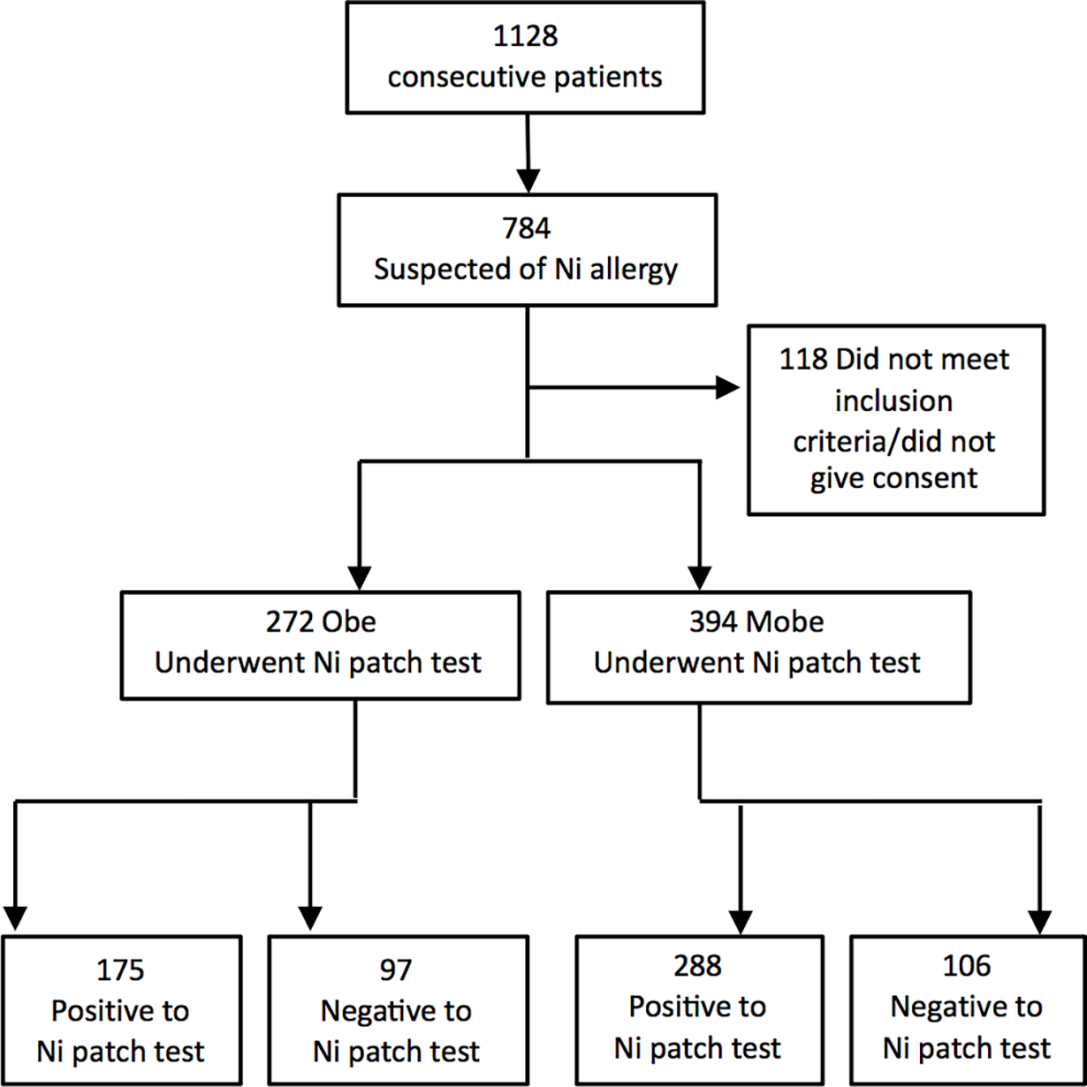
Flowchart of patient enrolment and stratification by BMI in Overweight and mildly obese (Obe) and MOrbidly Obese (MObe).

Overweight and mildly obese subjects frequently show different prevalence of comorbidities compared to morbidly obese patients, leading to a stratified risk of all-cause mortality (not increased in overweight and grade I obesity, increased in grade II and III)[19]. Weight can be itself an important confounding factor for metabolic assessments, and we therefore stratified the population in exam by BMI, subdividing it in a group of overweight and grade I obese patients (25<BMI<35) (Obe) and another of morbidly obese patients (> = 35) (MObe). Each of the groups was then subdivided according to Ni patch test results: positive or negative (Fig 1).

### Anthropometric measurements

Body weight and height were obtained between 8 and 10 a.m. in fasting subjects wearing light clothing and no shoes with an empty bladder. The same calibrated scale and stadiometer were used for all patients. Waist circumference was measured in the same instance at the midpoint between the lower rib margin and the iliac crest, the patients had their waist uncovered and were asked to stand with their feet close together and their weight equally distributed on each leg.

### Laboratory assessments

Blood samples were collected from fasting patients by venipuncture between 8 and 9 a.m. Samples were then transferred to the local laboratory and handled according to the local standards of practice. C Reactive Protein (CRP), Insulin, Glucose, Glycosilated Haemoglobin A1C (HbA1C), Insulin like Growth Factor 1 (IGF-1), GH [20], and routine laboratory tests were measured. HOmeostasis model assessment of insulin resistance (HOMA-IR) was calculated from fasting plasma insulin and glucose levels using the following formula: insulin (mIU/mL) x glucose (mg/dL) / 405 [21].

### Dynamic test

Patients showing low IGF-1 levels (<50 percentile for age and sex) together with organic disease or a history of head injury were tested for possible GH deficiency according to the AACE guidelines with GHRH (GHRH 1–29; Ferring, Italy; 1 μg/kg BW iv at time 0) plus Arginine (0.5 g/kg BW, L-ARG mono-hydrochloride by iv, from time 0 over 30 min) [22]. Written informed consent for the medical procedure was obtained from all patients. Blood samples were drawn from an indwelling catheter inserted in an antecubital vein at times 0, 30, 45, 60 min. All studies started between 8 and 9 AM, after an overnight fast. GH deficiency (GHD) was diagnosed according to AACE guidelines as a peak value below 4.8 ng/dL [22].

### Body composition

Body composition was measured by Dual X-Ray Absorptiometry (DXA) (QDR Discovery Acclaim, Hologic Inc., Waltham, MA) in fasting patients wearing light clothing and no shoes.

### Patch test

Patients with a clinical suspect of ACD or SNAS that met inclusion and exclusion criteria were patch tested with the Società Italiana Dermatologia Allergologica Professionale ed Ambientale (SIDAPA) baseline series (Lofarma S.p.A., Milano, Italy), that assess Ni sensitivity with a 5% Nickel Sulfate solution. Finn Chambers® (diameter, 8 mm; SmartPractice®, Phoenix, AZ, USA) on Scanpor® tape (Norgesplaster A/S, Vennesla, Norway) were applied and left on the back for 48 hours [23]. The readings were done on day 3 by a trained allergologist (Si.M.). For the patch test analysis, reactions “+” to “+++” were classified as positive, and negative and doubtful reactions as non-positive.

### Statistical analysis

Data are expressed as mean ± standard deviation (SD) if not otherwise stated. Normality was assessed with the Shapiro–Wilk test and variables were Log transformed when distribution was non-normal. Independent samples Student’s *t*-test and Mann-Whitney *u*-test were used to assess differences between groups. Differences between GH levels among groups upon dynamic testing were assessed by repeated measures analysis. Differences were considered statistically significant when p < .05. Statistical analysis was performed using GraphPad Prism Version 5.00 for Windows, GraphPad Software, San Diego California USA and SPSS Statistics for Windows, Version 20.0, Armonk, NY, USA: IBM Corp.

## Results

### Ni allergy in obese and overweight subjects

1128 obese and overweight outpatients accessing the High Specialization Center for the care of Obesity, Sapienza University of Rome, from 2010 to 2016 were evaluated. 69.5% (n = 784) reported complaints compatible with ACD or SNAS. Of these, 45.9% (n = 666) met inclusion and exclusion criteria and were therefore subjected to a 5% Ni Sulphate Patch test. The demographic, anthropometric, and clinical characteristics of the patients are shown in Table 1. Of the patients undergoing the diagnostic procedure, 69.5% (n = 463) resulted positive, the rest being negative, and no undetermined result was detected (Table 1). 71.1% (n = 432) of the women being tested were positive and 28.9% (n = 176) were negative, whereas male subjects tested positive in 53.45% of the cases (n = 31) and negative in 46.55% (n = 27). After screening through medical history and physical examination, 41% (463/1128) of the total number of obese patients accessing our center resulted allergic to Ni. Once stratified by BMI, Obe patients tested positive in 64.3% (175/272) of cases, whereas MObe were 73.1% (288/394) positive, more frequently than Obe patients (p = .016; Fig 1, Table 1).

**Table 1:** General characteristics of the population reporting at least two signs and symptoms of Allergic Contact Dermatitis or Systemic Nickel Allergy Syndrome and undergoing a Ni patch test analyzed as a whole population and stratified in Obe and MObe group.

Demographic, Anthropometric and Metabolic Parameters	Tested Population			Obe			MObe		
	(n = 666)			(n = 272)			(n = 394)		
						
	Mean±SD			Mean±SD			Mean±SD		
**Age (years)**	45	±	13	44,5	±	13,7	44,9	±	13,5
**Gender (%F)**	91,4			94,9			89,1*		
**Ni Patch test (% Positive)**	69,5			64,3			73,1*		
**Weight (kg)**	100,3	±	21,7	82,3	±	10,5	112,6	±	18,5*
**BMI (kg/m2)**	37,6	±	7,4	31,0	±	2,7	42,2	±	6,1*
**Waist circumference (cm)**	116,8	±	16,3	103,8	±	9,7	125,4	±	14,5*
**Fat mass (%)**	40,3	±	5,8	37,9	±	5,3	42,1	±	5,5*
**Lean mass (%)**	59,7	±	5,8	62,1	±	5,3	57,9	±	5,5*
**Total Cholesterol (mg/dL)**	199,1	±	40,4	199,3	±	43,7	198,8	±	38,1
**LDL-C (mg/dL)**	123,2	±	33,2	122,2	±	35,7	123,8	±	31,4
**HDL-C (mg/dL)**	50,1	±	13,2	52,6	±	13,8	48,5	±	12,5*
**Triglycerides (mg/dL)**	132,2	±	94,5	118,8	±	75,4	141,2	±	104,8*
**Glucose (mg/dL)**	97,2	±	22,3	93,4	±	16,1	99,8	±	25,3*
**Insulin (mIU/L)**	19,2	±	15,8	14,9	±	12,5	22,2	±	17,2*
**HOMA-IR**	4,8	±	4,6	3,6	±	3,4	5,6	±	5,1*
**Creatinine (mg/dL)**	,8	±	,2	0,8	±	0,2	0,8	±	0,2
**CRP (mg/L)**	,7	±	,7	0,4	±	0,6	0,9	±	0,9*
**HbA1C (%)**	5,6	±	,9	5,4	±	0,8	5,7	±	0,9*
**GH (ng/ml)**	,9	±	1,5	1,3	±	2,0	0,6	±	1,0*
**IGF-1 (ng/ml)**	176,8	±	99,0	197,5	±	106,5	163,0	±	91,0*

Abbreviations: SD, Standard Deviation; F, Female; Ni, Nickel; BMI, Body Mass Index; LDL-C, Low Density Lipoprotein Cholesterol; HDL-C, High Density Lipoprotein Cholesterol; HOMA-IR, HOmeostasis Model Assessment-Insulin Resistance; HbA1C, Hemoglobin A1C; CRP, C Reactive Protein; GH, Growth Hormone, IGF-1, Insulin Like Factor -1.

*compared to Obe, p < .05.

### Metabolic status

Ni allergy was associated with significantly higher BMI and worse body composition. Moreover, allergic patients showed worse glucose homeostasis with higher insulin, HOMA-IR and HbA1C levels. In addition to this, the same patients had increased inflammation as shown by higher CRP levels (Table 2).

**Table 2:** Characteristics of the general population reporting at least two signs and symptoms of Allergic Contact Dermatitis or Systemic Nickel Allergy Syndrome undergoing a Ni patch test.

Demographic, Anthropometric and Metabolic Parameters	Ni Allergic			Non Ni Allergic		
	Mean±SD			Mean±SD		
**Age (years)**	45	±	13	44	±	14
**Gender (%F)**	92,8			84,7*		
**Weight (kg)**	101,3	±	22,2	97,7	±	20,3
**BMI (kg/m2)**	38,1	±	7,7	36,3	±	6,6**
**Waist circumference (cm)**	116,9	±	17,46	116,9	±	15,7
**Fat mass (%)**	40,7	±	5,5	39,4	±	6,5*
**Lean mass (%)**	59,3	±	5,5	60,6	±	6,5*
**Total Cholesterol (mg/dL)**	199	±	40	199	±	41
**LDL-C (mg/dL)**	123	±	33	124	±	34
**HDL-C (mg/dL)**	50	±	13	51	±	14
**Triglycerides (mg/dL)**	136	±	103	123	±	72
**Glucose (mg/dL)**	98	±	24	95	±	18
**Insulin (mIU/L)**	20	±	16	17	±	14**
**HOMA-IR**	5,1	±	5	4	±	3,6**
**Creatinine (mg/dL)**	0,8	±	0,2	,8	±	,2
**CRP (mg/L)**	0,8	±	0,8	,6	±	,6***
**HbA1C (%)**	5,6	±	0,9	5,5	±	,8*
**GH (ng/ml)**	0,8	±	1,3	1,1	±	1,9
**IGF-1 (ng/ml)**	170	±	100	193	±	95**
**GH Deficiency (% Positive)**	35,1			12,5**		

Differences between patients testing positive or negative to a Ni patch test are shown. Abbreviations: Ni, Nickel; SD, Standard Deviation; F, Female; BMI, Body Mass Index; LDL-C, Low Density Lipoprotein Cholesterol; HDL-C, High Density Lipoprotein Cholesterol; HOMA-IR, HOmeostasis Model Assessment-Insulin Resistance; HbA1C, Hemoglobin A1C; CRP, C Reactive Protein; GH, Growth Hormone, IGF-1, Insulin Like Factor -1.

* p < .05

** p < .01

*** p < .001

Menopausal status and age, factors that could easily affect metabolic health, were not significantly different between Obe and MObe groups (35.5% vs 33.6% menopausal female, p = .67; 44.5±13.7 vs 44.4±13.2 age, p = .88). However, gender distribution differed significantly, females being more represented in the Obe group (94.9 vs 89.1, p = .01).

Among Obe patients, mean BMI was not significantly different between the positive and negative group, but body composition was worse in allergic patients (i.e lean mass was lower in the positive group). None of the assessed metabolic parameters were significantly different other than CRP that was increased in allergic patients (Table 3).

**Table 3:** Characteristics of the population with at least two signs and symptoms of Allergic Contact Dermatitis or Systemic Nickel Allergy Syndrome undergoing a Ni patch test stratified by BMI in Overweight and mildly obese (Obe) and Morbidly Obese (MObe).

Demographic, Anthropometric and Metabolic Parameters	Obe						MObe					
	Ni Allergic			Non Ni allergic			Ni Allergic			Non Ni allergic		
	Mean±SD			Mean±SD			Mean±SD			Mean±SD		
**Age (years)**	45	±	13	42	±	14	45	±	13	44	±	14
**Gender (%F)**	97,7			87,4			89,7			82,2		
**Weight (kg)**	81,8	±	11,0	82,8	±	9,3	113,2	±	18,6	111,0	±	18,1
**BMI (kg/m2)**	30,9	±	2,7	30,8	±	2,6	42,6	±	6,3	41,9	±	5,1
**Waist circumference (cm)**	103,1	±	10,24	104,5	±	9,06	126,5	±	14,83	124,3	±	14,07
**Fat mass (%)**	38,5	±	4,8	36,9	±	5,9	42,2	±	5,3	41,9	±	6,0
**Lean mass (%)**	61,5	±	4,8	63,1	±	5,9*	57,8	±	5,3	58,1	±	6,0
**Total Cholesterol (mg/dL)**	201	±	44	195	±	42	197	±	37	202	±	39
**LDL-C (mg/dL)**	122	±	37	120	±	33	122	±	30	126	±	33
**HDL-C (mg/dL)**	52	±	13	52	±	14	47	±	12	50	±	13
**Triglycerides (mg/dL)**	122	±	83	111	±	58	143	±	112	133	±	81
**Glucose (mg/dL)**	94	±	18	91	±	10	100	±	26	98	±	22
**Insulin (mIU/L)**	15,2	±	11,7	14,3	±	13,8	23,3	±	18,0	18,9	±	14,2*
**HOMA-IR**	3,7	±	3,4	3,3	±	3,3	6,0	±	5,5	4,6	±	3,8*
**Creatinine (mg/dL)**	,75	±	,14	,78	±	,17	,76	±	,18	,77	±	,14
**CRP (mg/L)**	,46	±	,66	,38	±	,53*	,96	±	,90	,74	±	,65*
**HbA1C (%)**	5,4	±	,6	5,4	±	1,0	5,8	±	,9	5,5	±	,6
**GH (ng/ml)**	1,06	±	1,66	1,69	±	2,57	,60	±	,99	,70	±	,92
**IGF-1 (ng/ml)**	187,0	±	108,9	214,6	±	99,5*	158,3	±	92,2	176,3	±	88,3*
**GH Deficit (% Positive)**	21,6			15			40,2			11,1*		

Differences between patients testing positive or negative to a Ni patch test are shown. Abbreviations: Ni, Nickel; SD, Standard Deviation; F, Female; BMI, Body Mass Index; LDL-C, Low Density Lipoprotein Cholesterol; HDL-C, High Density Lipoprotein Cholesterol; HOMA-IR, HOmeostasis Model Assessment-Insulin Resistance; HbA1C, Hemoglobin A1C; CRP, C Reactive Protein; GH, Growth Hormone, IGF-1, Insulin Like Factor -1

* p < .05

** p < .01.

Among MObe patients, mean BMI was not significantly different between the positive and negative group, neither were anthropometric parameters, but there were striking differences regarding glucose homeostasis parameters. Positive patients showed in fact significantly higher fasting insulin and HOMA-IR. Moreover, CRP was significantly increased in positive patients (Table 3).

### GH IGF1 axis

**Fig 2:**
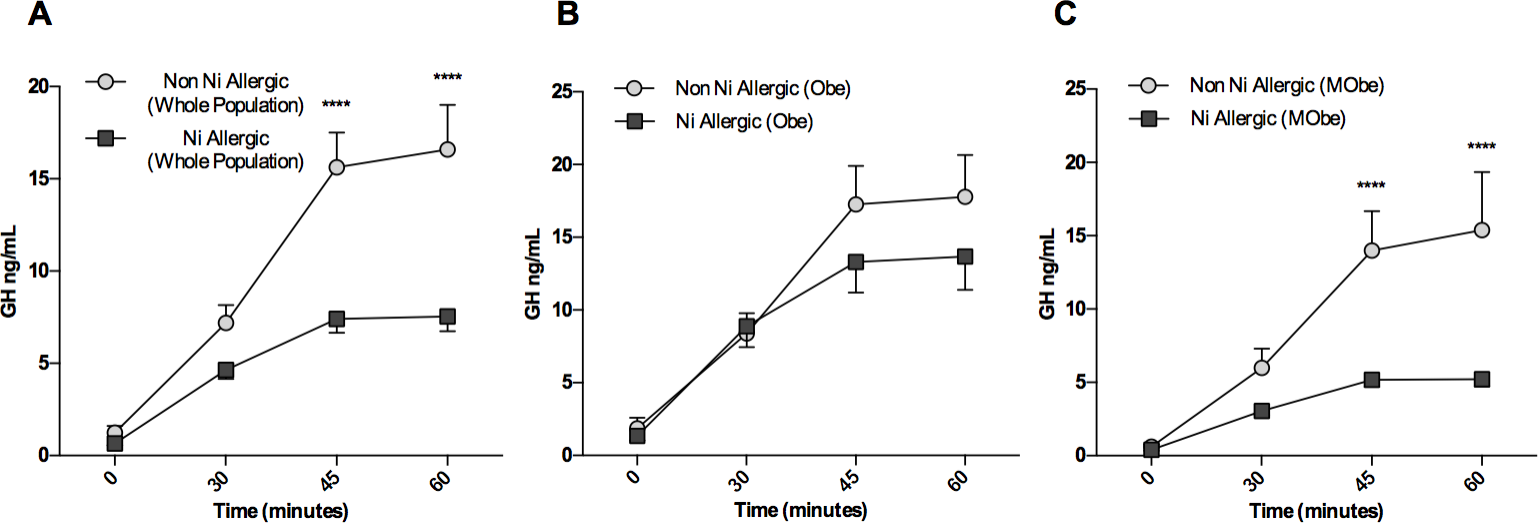
GHRH+Arginine Dynamic Test. **A.** Non-stratified population undergoing a GHRH+Arginine Dynamic Test. Time 45 and 60 are significantly different between patients who are allergic to Ni and patients who are not, with allergic patients showing a significantly blunted response. **B.** Obe patients show the same trend as the non-stratified population but fail to show a significant difference at any time point. **C.** MObe patients confirm the same significantly blunted response shown by the general population at time point 45 and 60. ****, p < .0001.

Overall, Ni allergy was associated with impaired GH-IGF-1 axis function. In fact, allergic patients showed significantly lower baseline IGF-1 and blunted GH dynamic response upon GHRH+Arginine stimulus compared to non-allergic ones even when controlling for BMI, age and HOMA-IR (Table 2, Fig 2A). GHD diagnosed according to AACE guidelines that take into consideration BMI as a known condition of impaired GH response [22] was more frequent in patch test positive patients (47/134, 35.1%) than in patch test negative ones (5/40, 12.5%, p = .006) (Table 2).

Upon stratification by BMI, the impairment in the GH-IGF-1 axis function was not present in allergic Obe patients, who failed to show significantly GH (Table 3), or a blunted response to dynamic testing (Fig 2B). GHD diagnosis was not more frequent in Obe allergic patients (Table 3). Of note, baseline IGF-1 was significantly lower in allergic patients.

Conversely, allergic MObe patients showed, on top of significantly lower baseline IGF-1 levels, a marked decrease in the dynamic response compared to those who tested negative to the Ni patch test (Table 3, Fig 2C), that upon repeated measures testing remained significant even after controlling for HOMA-IR and BMI baseline values. GHD diagnosis was significantly more frequent in allergic MObe compared to non-allergic ones (Table 3, p = .01).

## Discussion

ACD and SNAS are complex conditions whose pathogenesis is yet to be fully elucidated. It has been suggested that an innate immune response, type I hypersensitivity and type IV immune reaction are responsible for cutaneous flare-ups [24, 25], whereas circulating immune complexes (Type III reaction) and non-specific cytokine release may be in turn potentially responsible for the generalized symptoms of SNAS [26], leading altogether to enhanced inflammatory response. Unequivocal evidence produced in the past decades shows that low grade inflammation plays an important role in the pathogenesis of metabolic syndrome, where the activation of specific pathways leading to adipocyte dysfunction seems to lie at the bottom of the question[27–29]. Moreover, evidence from recent preclinical studies report that oxidative stress, increased membrane permeability, reduced number of mitochondria and decreased expression of Uncoupling Protein 1 (UCP-1) and other genes related to Brown Adipose Tissue (BAT) may be other possible links between Ni exposure and metabolic homeostasis impairment that are unrelated to that of chronic inflammation [4–9]. Reducing dietary Ni intake substantially ameliorates systemic Ni allergy symptoms [30, 31]. Moreover, Lusi et al. have recently reported that a low Ni diet is able to induce weight loss in patients allergic to Ni, pointing in the same direction as pre-clinical studies [15].

Our study partially confirms what previously suggested from Lusi et al. that obese patients seem to be more frequently allergic to Ni compared to the general population. The authors have reported that 59.7% of an overweight population had Ni allergy. The difference we see in frequency compared to their case series may be due to their small sample size and/or our selection of the patients undergoing a Ni patch test. Interestingly, we see that obese male subjects appear to be more prone of females to be sensitized, conversely to what usually observed in the general population, where male subjects are by far less allergic than women [3]. However, we had a small number of male subjects in our population and our finding should be therefore confirmed in a more numerous sample. The reason of such a gap in male versus female representation possibly lies in several factors. First, it is acknowledged that female obese patients more frequently consider obesity as a disease and therefore seek medical assistance for it [32–34]. As our population accessed a specialized center for obesity care we initially enrolled more female than male patients. Moreover, several signs and symptoms possibly pointing at ACD or SNAS such as bloating, fatigue and headache are more frequently reported by female individuals, most likely for a number of reasons including psychological and cultural ones [35]. A major limitation of our study is the initial screening that the patients underwent in order to be tested for Ni allergy, that may have created a selection bias possibly underestimating the total and gender specific prevalence, as patients with no suspect were not assessed at all. It should be noted that the estimated daily intake of Ni in Italy is higher than most other European countries and the US [36]. This may be an additional reason why our prevalence data is much higher than that estimated in the general population of other countries.

We report that patients who are allergic to Ni are heavier, have worse body composition, impaired glucose homeostasis, and increased inflammation, suggesting a role of Ni in such differences. Upon stratification by BMI, Obe only show differences regarding body composition between allergic and non-allergic patients, with no metabolic impairment. On the contrary, MObe who are allergic to Ni have worse glucose homeostasis compared to non-allergic MObe patients. Increased inflammation is present in both Obe and MObe allergic patients. Based on our observations it is likely that low grade inflammation and direct toxicity induced by Ni are additive risk factors for metabolic impairment together with others and obesity itself, thus the worse status of patients allergic to Ni where both direct toxicity and immune mediated effects take place compared to non-allergic patients where direct toxicity is probably the only Ni related risk factor and where a better handling of Ni accumulation may exist [37]. Moreover, we believe that MObe patients have a more pronounced metabolic impairment compared to Obe patients because of other factors contributing to their health condition.

We also report an impaired function in the GH-IGF-1 axis in all Ni allergic patients. Upon stratification, allergic MObe confirm such impairment compared to non-allergic MObe: this can be partially explained by the significantly different glucose homeostasis status of this group, where insulin is higher in the allergic group. In fact, solid evidence in literature suggests that hyperinsulinism suppresses GH production via direct pituitary action on GH synthesis and release and indirect action via modulation of hypothalamic function, alterations in the availability of IGF-I, and/or suppression of circulating ghrelin levels [38–42]. Confirming this, Obe subjects do not show a significantly different performance upon dynamic testing, compatible with the unimpaired glucose homeostasis. However, when controlling for baseline HOMA IR and BMI, time points 45’ and 60’ remain significantly different in allergic compared to non-allergic MObe, suggesting other concomitant factors accounting for these profoundly different responses. Of note, *in vitro* studies have previously suggested a possible disrupting role of Ni in pituitary function through inhibition of Calcium uptake or redistribution and consequent reduced secretion of prolactin and GH [10–12]. However, *in vivo* evidence is lacking, and we cannot therefore infer that this may be an additional mechanism through which the GH-IGF-1 axis is impaired in allergic MObe patients.

In conclusion, our study suggests an increased presence of Ni allergy in Italian obese patients and a possible link between obesity, hormonal dysregulation and Ni allergy/exposure. Toxic and immune-mediated effects of Ni may synergistically play a role in the genesis of obesity and hormonal impairment, but several questions still need to be answered in order to understand the full picture and the mechanisms via which Ni may exert such metabolic effects in human subjects.

## Abbreviations

Ni: Nickel
ACD: Allergic Contact Dermatitis
SNAS: Systemic Nickel Allergy Syndrome
SCD: Systemic Contact Dermatitis
EFSA: European Food Safety Authority
CRP: C Reactive Protein
HbA1C: Hemoglobin A1C
IGF-1: Insulin like Growth Factor 1
GH: Growth Hormone
HOMA-IR: HOmeostasis Model Assessment of Insulin Resistance
GHRH: Growth Hormone Releasing Hormone
GHD: Growth Hormone Deficiency
DXA: Dual X-Ray Absorptiometry
SIDAPA: Società Italiana Dermatologia Allergologica Professionale ed Ambientale
SD: Standard Deviation
MAPK: Mitogen Activated Protein Kinase
JNK: c-Jun N-terminal Kinase
PKC: Protein Kinase C
IRS: Insulin Receptor Substrate
COX-1: CycloOxigenase-1
UCP-1: Uncoupling Protein-1
BAT: Brown Adipose Tissue
F: Female
BMI: Body Mass Index
LDL-C: Low Density Lipoprotein Cholesterol
HDL-C: High Density Lipoprotein Cholesterol.

## References

[1] Obesity and Overweight Factsheet June 2016 [08/04/2017]. Available from: http://www.who.int/mediacentre/factsheets/fs311/en/.

[2] Saito M, Arakaki R, Yamada A, Tsunematsu T, Kudo Y, Ishimaru N. Molecular Mechanisms of Nickel Allergy. Int J Mol Sci. 2016;17(2). 10.3390/ijms17020202 ; PubMed Central PMCID: PMCPMC4783936. 26848658 PMC4783936

[3] Ahlstrom MG, Thyssen JP, Menne T, Johansen JD. Prevalence of nickel allergy in Europe following the EU Nickel Directive—a review. Contact Dermatitis. 2017 10.1111/cod.12846 . 28730624

[4] Xu X, Rao X, Wang TY, Jiang SY, Ying Z, Liu C, et al Effect of co-exposure to nickel and particulate matter on insulin resistance and mitochondrial dysfunction in a mouse model. Part Fibre Toxicol. 2012;9:40 10.1186/1743-8977-9-40 ; PubMed Central PMCID: PMCPMC3545913. 23126276 PMC3545913

[5] Lin YC, Lian IB, Kor CT, Chang CC, Su PY, Chang WT, et al Association between soil heavy metals and fatty liver disease in men in Taiwan: a cross sectional study. BMJ Open. 2017;7(1):e014215 10.1136/bmjopen-2016-014215 ; PubMed Central PMCID: PMCPMC5278238. 28115335 PMC5278238

[6] Gupta S, Ahmad N, Husain MM, Srivastava RC. Involvement of nitric oxide in nickel-induced hyperglycemia in rats. Nitric Oxide. 2000;4(2):129–38. 10.1006/niox.2000.0278 . 10835293

[7] Dzugkoeva FS, Mozhaeva IV, Dzugkoev SG, Margieva OI, Tedtoeva AI, Otiev MA. Oxidative Stress and Biochemical Markers of Endothelial Dysfunction and Organ Damage under Conditions of Experimental Nonferrous Metal Intoxication. Bull Exp Biol Med. 2016;162(2):199–202. 10.1007/s10517-016-3575-z . 27909964

[8] Das KK, Das SN, Dhundasi SA. Nickel, its adverse health effects & oxidative stress. Indian J Med Res. 2008;128(4):412–25. . 19106437

[9] Chen YW, Yang CY, Huang CF, Hung DZ, Leung YM, Liu SH. Heavy metals, islet function and diabetes development. Islets. 2009;1(3):169–76. 10.4161/isl.1.3.9262 . 21099269

[10] Carlson HE. Inhibition of prolactin and growth hormone secretion by nickel. Life Sci. 1984;35(17):1747–54. . 6090848 10.1016/0024-3205(84)90271-6

[11] Dormer RL, Kerbey AL, McPherson M, Manley S, Ashcroft SJ, Schofield JG, et al The effect of nickel on secretory systems. Studies on the release of amylase, insulin and growth hormone. Biochem J. 1974;140(2):135–42. ; PubMed Central PMCID: PMCPMC1167985. 4375956 10.1042/bj1400135 PMC1167985

[12] LaBella FS, Dular R, Lemon P, Vivian S, Queen G. Prolactin secretion is specifically inhibited by nickel. Nature. 1973;245(5424):330–2. . 4357307 10.1038/245330a0

[13] Jacob SE, Goldenberg A, Pelletier JL, Fonacier LS, Usatine R, Silverberg N. Nickel Allergy and Our Children's Health: A Review of Indexed Cases and a View of Future Prevention. Pediatr Dermatol. 2015;32(6):779–85. 10.1111/pde.12639 . 26212605

[14] Christensen OB, Moller H. External and internal exposure to the antigen in the hand eczema of nickel allergy. Contact Dermatitis. 1975;1(3):136–41. . 797515 10.1111/j.1600-0536.1975.tb05354.x

[15] Lusi EA, Di Ciommo VM, Patrissi T, Guarascio P. High prevalence of nickel allergy in an overweight female population: a pilot observational analysis. PLoS One. 2015;10(3):e0123265 10.1371/journal.pone.0123265 ; PubMed Central PMCID: PMCPMC4379055. 25822975 PMC4379055

[16] Rietschel RL, Fowler JF, Warshaw EM, Belsito D, DeLeo VA, Maibach HI, et al Detection of nickel sensitivity has increased in North American patch-test patients. Dermatitis. 2008;19(1):16–9. . 18346391

[17] Chain EPoCitF. Scientific Opinion on the risks to public health related to the presence of nickel in food and drinking water. EFSA Journal. 2015;13(2):4002–n/a. 10.2903/j.efsa.2015.4002 PMC13092915 42016839

[18] Di Tola M, Amodeo R, Marino M, Tabacco F, Casale R, Bove M, et al Peripheral Blood Lymphocyte Typing as a Useful Tool to Objectify the Oral Mucosa Patch Test in the Diagnosis of Allergic Contact Mucositis to Nickel. Biological Trace Element Research. 2014;159(1):81–6. 10.1007/s12011-014-9991-x 24789478

[19] Flegal KM, Kit BK, Orpana H, Graubard BI. Association of all-cause mortality with overweight and obesity using standard body mass index categories: a systematic review and meta-analysis. JAMA. 2013;309(1):71–82. Epub 2013/01/03. 10.1001/jama.2012.113905 ; PubMed Central PMCID: PMCPMC4855514. 23280227 PMC4855514

[20] Seetho IW, Wilding JP. How to approach endocrine assessment in severe obesity? Clin Endocrinol (Oxf). 2013;79(2):163–7. 10.1111/cen.12256 . 23734868

[21] Matthews DR, Hosker JP, Rudenski AS, Naylor BA, Treacher DF, Turner RC. Homeostasis model assessment: insulin resistance and beta-cell function from fasting plasma glucose and insulin concentrations in man. Diabetologia. 1985;28(7):412–9. . 3899825 10.1007/BF00280883

[22] Cook DM, Yuen KC, Biller BM, Kemp SF, Vance ML, American Association of Clinical E. American Association of Clinical Endocrinologists medical guidelines for clinical practice for growth hormone use in growth hormone-deficient adults and transition patients—2009 update. Endocr Pract. 2009;15 Suppl 2:1–29. 10.4158/EP.15.S2.1 . 20228036

[23] Lachapelle J-M, Maibach HI. Patch testing and prick testing: A practical guide official publication of the ICDRG: Springer Science & Business Media; 2012.

[24] Buyukozturk S, Gelincik A, Unal D, Demirturk M, Celik DD, Erden S, et al Oral nickel exposure may induce Type I hypersensitivity reaction in nickel-sensitized subjects. Int Immunopharmacol. 2015;26(1):92–6. 10.1016/j.intimp.2015.03.012 . 25797346

[25] Schmidt M, Goebeler M. Nickel allergies: paying the Toll for innate immunity. J Mol Med (Berl). 2011;89(10):961–70. 10.1007/s00109-011-0780-0 . 21698426

[26] Nijhawan RI, Molenda M, Zirwas MJ, Jacob SE. Systemic contact dermatitis. Dermatol Clin. 2009;27(3):355–64, vii. 10.1016/j.det.2009.05.005 . 19580929

[27] Hotamisligil GS. Inflammation and metabolic disorders. Nature. 2006;444(7121):860–7. 10.1038/nature05485 . 17167474

[28] Romeo GR, Lee J, Shoelson SE. Metabolic syndrome, insulin resistance, and roles of inflammation—mechanisms and therapeutic targets. Arterioscler Thromb Vasc Biol. 2012;32(8):1771–6. 10.1161/ATVBAHA.111.241869 ; PubMed Central PMCID: PMCPMC4784686. 22815343 PMC4784686

[29] Saraswathi V, Ramnanan CJ, Wilks AW, Desouza CV, Eller AA, Murali G, et al Impact of hematopoietic cyclooxygenase-1 deficiency on obesity-linked adipose tissue inflammation and metabolic disorders in mice. Metabolism. 2013;62(11):1673–85. 10.1016/j.metabol.2013.07.007 ; PubMed Central PMCID: PMCPMC4845736. 23987235 PMC4845736

[30] Braga M, Quecchia C, Perotta C, Timpini A, Maccarinelli K, Di Tommaso L, et al Systemic nickel allergy syndrome: nosologic framework and usefulness of diet regimen for diagnosis. Int J Immunopathol Pharmacol. 2013;26(3):707–16. 10.1177/039463201302600314 . 24067467

[31] Antico A, Soana R. Chronic allergic-like dermatopathies in nickel-sensitive patients. Results of dietary restrictions and challenge with nickel salts. Allergy Asthma Proc. 1999;20(4):235–42. . 10476323 10.2500/108854199778338991

[32] Bunt SNW, Merelle SYM, Steenhuis IHM, Kroeze W. Predictors of need for help with weight loss among overweight and obese men and women in the Netherlands: a cross-sectional study. BMC Health Serv Res. 2017;17(1):819 Epub 2017/12/14. 10.1186/s12913-017-2759-1 ; PubMed Central PMCID: PMCPMC5728017. 29233134 PMC5728017

[33] Tol J, Swinkels IC, De Bakker DH, Veenhof C, Seidell JC. Overweight and obese adults have low intentions of seeking weight-related care: a cross-sectional survey. BMC Public Health. 2014;14:582 Epub 2014/06/12. 10.1186/1471-2458-14-582 ; PubMed Central PMCID: PMCPMC4085466. 24916037 PMC4085466

[34] Wolfe BL, Smith JE. Different strokes for different folks: why overweight men do not seek weight loss treatment. Eat Disord. 2002;10(2):115–24. Epub 2006/07/26. 10.1080/10640260290081687 . 16864252

[35] Ihlebaek C, Eriksen HR, Ursin H. Prevalence of subjective health complaints (SHC) in Norway. Scand J Public Health. 2002;30(1):20–9. Epub 2002/04/04. . 11928829

[36] Pizzutelli S. Systemic nickel hypersensitivity and diet: myth or reality? Eur Ann Allergy Clin Immunol. 2011;43(1):5–18. . 21409856

[37] Patriarca M, Lyon TD, Fell GS. Nickel metabolism in humans investigated with an oral stable isotope. Am J Clin Nutr. 1997;66(3):616–21. 10.1093/ajcn/66.3.616 . 9280182

[38] Grottoli S, Gauna C, Tassone F, Aimaretti G, Corneli G, Wu Z, et al Both fasting-induced leptin reduction and GH increase are blunted in Cushing's syndrome and in simple obesity. Clin Endocrinol (Oxf). 2003;58(2):220–8. . 12580939 10.1046/j.1365-2265.2003.01699.x

[39] Lanzi R, Luzi L, Caumo A, Andreotti AC, Manzoni MF, Malighetti ME, et al Elevated insulin levels contribute to the reduced growth hormone (GH) response to GH-releasing hormone in obese subjects. Metabolism. 1999;48(9):1152–6. . 10484056 10.1016/s0026-0495(99)90130-0

[40] Maccario M, Grottoli S, Procopio M, Oleandri SE, Rossetto R, Gauna C, et al The GH/IGF-I axis in obesity: influence of neuro-endocrine and metabolic factors. Int J Obes Relat Metab Disord. 2000;24 Suppl 2:S96–9. . 10997620 10.1038/sj.ijo.0801289

[41] Scacchi M, Pincelli AI, Cavagnini F. Growth hormone in obesity. Int J Obes Relat Metab Disord. 1999;23(3):260–71. . 10193871 10.1038/sj.ijo.0800807

[42] Luque RM, Kineman RD. Impact of obesity on the growth hormone axis: evidence for a direct inhibitory effect of hyperinsulinemia on pituitary function. Endocrinology. 2006;147(6):2754–63. 10.1210/en.2005-1549 . 16513828

